# Simulation of Slip-Oxidation Process by Mesh Adaptivity in a Cohesive Zone Framework

**DOI:** 10.3390/ma14133509

**Published:** 2021-06-23

**Authors:** Michal Sedlak Mosesson, Bo Alfredsson, Pål Efsing

**Affiliations:** 1 Department of Engineering Mechanics, Royal Institute of Technology KTH, SE-100 44 Stockholm, Sweden; alfred@kth.se (B.A.); efsing@kth.se (P.E.); 2 Ringhals AB, SE-43285 Väröbacka, Sweden

**Keywords:** cohesive zone model, fracture mechanics, diffusion, oxide film, slip oxidation

## Abstract

Adaptive oxide thickness was developed in a cohesive element based multi-physics model including a slip-oxidation and diffusion model. The model simulates the intergranular stress corrosion cracking (IGSCC) in boiling water reactors (BWR). The oxide thickness was derived from the slip-oxidation and updated in every structural iteration to fully couple the fracture properties of the cohesive element. The cyclic physics of the slip oxidation model was replicated. In the model, the thickness of the oxide was taken into consideration as the physical length of the cohesive element. The cyclic process was modelled with oxide film growth, oxide rupture, and re-passivation. The model results agreed with experiments in the literature for changes in stress intensity factor, yield stress representing cold work, and environmental factors such as conductivity and corrosion potential.

## 1. Introduction

When assessing nuclear power plant life, stress corrosion cracking (SCC) plays an important role. Stress corrosion cracking in nuclear power plants is well phenomenologically recognized and still heavily researched [1,2]. Despite this, SCC is not fully understood due to its complicated multiphysical nature. There are many different damage mechanisms behind SCC. The model category considered in the current work, is denoted slip-dissolution models [3,4] where the anodic dissolution is very high at the crack tip compared to the sides of the crack, which creates a growing sharp crack. The oxide created by the oxidation process is ruptured by the large plastic strains at the tip. This process is usually cyclic: anodic dissolution, oxide build-up and oxide rupture. The crack tip strain rate is often not well captured by the slip-dissolution models. The deficiency has been addressed [5,6] but still with limited success, due to the difficulties of resolving the localized dislocation movements. There are a few models with both full crack tip mechanism and oxidation kinetics at the crack tip [7]. There is also a model with node release as the fracture mechanism and remesh algorithm to handle the oxide growth, by Shoji et al. [8]. More phenomenological approaches are available with couplings between fracture mechanics, diffusion and corrosion. In Couvant et al. [9] the diffusion acts as a phenomenological corrosion, while in Sedlak et al. [10] the definition of corrosion degradation is phenomenological. There are also fully discrete structural models by Jivkov et al. [11,12]. The extended finite element method (XFEM) has also been used to show the effect of SCC [13].

**Figure 1:**
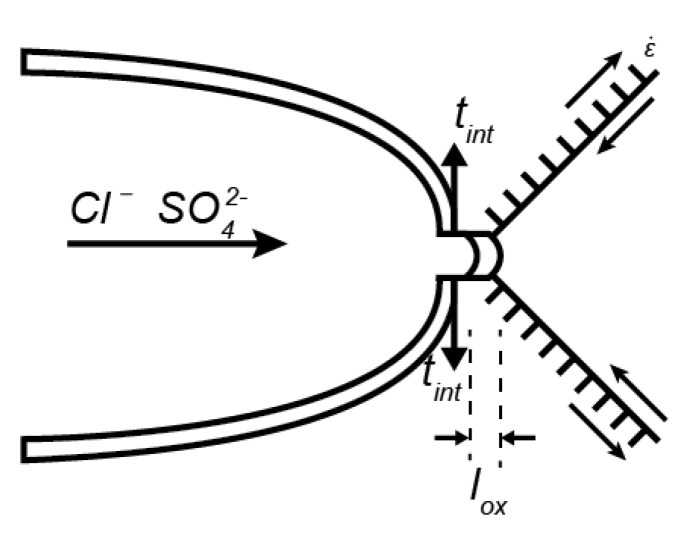
Schematic figure of the bulk material undergoing dislocation movement and diffusion of aggressive ions.

The present model is simulating the slip-dissolution mechanism. The aggressive ions diffused to the crack tip where they act as a catalyst to slow down the repassivation rate of the oxide film. At the crack tip the localized anodic dissolution occurred until an oxide film was produced to repassivate the corrosion process. Due to the constant stresses applied, the oxide film ruptured, and new virgin material was exposed to be dissolved and finally repassivated. This process was consequently repeated, see Figure 1. The environment considered was in the boiling water reactor (BWR) under normal water chemistry (NWC), containing approximately 200 ppb oxidant (O_2_ + H_2_O_2_) in the studied recirculation piping [14]. Considering the high temperature and the low amount of aggressive ions, SCC was assumed to be intergranular and the material considered was austenitic stainless steel in the 304 and 316L series.

The previous computational models lack the ability of efficiently track the oxide growth and rupture process including environmental influence. Here the oxide growth was tracked with an adaptive mesh framework. The model was multi-physical, consisting of a cohesive zone model (CZM) [15,16,17] to describe the fracture process, using the slip-oxidation model by Ford et al. [4] to describe the dissolution at the crack tip under the influence of impurities as ${\mathrm{Cl}}^{-}$ and ${\mathrm{SO}}_{4}^{2-}$, applying Fick’s second law to describe the diffusion of the aggressive anions to the crack tip.

A demanding computational part is the unknown (prior to simulations) oxide thickness *l_ox_* and the chained cyclic process off the oxides. To simulate the rupture process of the oxide during the slip-dissolution process conventional methods, as diffusion-based SCC or using gauss points could not describe every single oxide rupture. Instead mesh adaptivity was introduced, where the degradation process for every oxide was in a single cohesive element and the length was controlled by the growth of the oxide.

The surrounding material were describing with elastic-plastic finite element (FE) as a continuum, not considering grain structure or grain orientation. The crack was assumed to propagate between the grains as intergranular stress corrosion cracking (IGSCC). The cohesive model was pioneered by Barenblatt [18] and Dugdale [19]; later, it was put into a computational concept by Hillerborg [20]. The CZM describes the fracture process by introducing a traction separation law (TSL), which is the relationship between closing force and the separation. The TSL by Park et al. [21], called the PPR model, was implemented in the CZM in combination with the degradation feature implemented by Sedlak et al. [22]. The combination was used to change the fracture properties from that of the virgin bulk material to that of the oxide.

Fick’s second law was implemented to assess the diffusion of the aggressive ions to the crack tip, assuming long cracks made the electrochemical migration and convection low compared to the diffusion in the mass conservation equation. Therefore, the migration convection was excluded. The species considered to diffuse were ${\mathrm{Cl}}^{-}$ and ${\mathrm{SO}}_{4}^{2-}$ [14,23]. The diffusivity was linearly dependent on the cohesive element damage, which also created a boundary.

The crack tip electrochemistry was implemented by a slip-oxidation model at the crack-tip [4]. The model was based on Faraday’s law. Here, the current density was used and coupled with the damage process in the cohesive element. The growth of the passivation film was implemented by an adaptive control of the nodes in the CZM and the fracture properties were degraded with the PPR model with degradation capabilities.

In NWC, the conductivity and corrosion potential play important roles together with stress intensity for predicting crack growth. Therefore, the purpose was to create a coupled model which can predict the crack growth in NWC conditions. Additionally, an important factor behind SCC is cold work [20]. The effect of cold work has been shown experimentally by many researchers [24,25,26,27,28,29], and a goal was to predict the coupled effect from both environment and cold work.

## 2. Model

**Figure 2:**
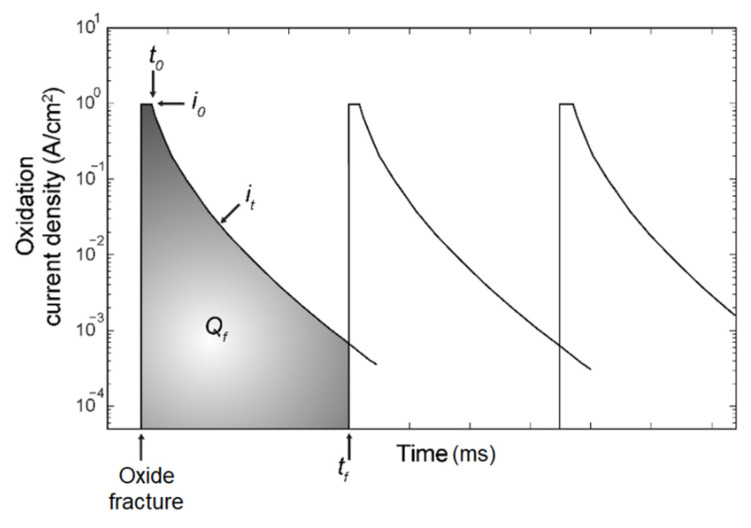
Schematic figure of the oxide current density with respect to time.

The electrochemical model at the crack tip was set up with the slip-oxidation model by Ford [4,30]. The transport of species to the crack tip was based on the diffusion model with the assumption of a preexisting long crack, compared to relevant sizes in the crack tip area such as oxide thickness and plastic zone. The slip-oxidation model is based on Faraday’s relationship between metal transformation (M^+^) to oxide and the oxide charge density. The slip-oxidation model is described as

(1) $$\frac{da}{dt}=\frac{M}{z\rho F}i$$

where *a* is the crack length, *M* is the atomic weight of the crack tip metal, *z* is the number of electrons exchanged from an atom to the metal, *ρ* is the density of the crack tip metal, *F* is the Faraday’s constant, and *i* is the current density, defined as

(2) $$i=i_{0}{\left(\frac{t}{t_{0}}\right)}^{-m}\;\;t_{0}<t<t_{f}$$

where $i_{0}$ is the initial current density, $t_{0}$ is the time for repassivation activation, and *m* is the decay slope, and the time between ruptures of the oxide film, or fracture time, is denoted as $t_{f}$. The cyclic behavior of the current density *i* is shown in Figure 2, as the film ruptures the dissolution is at its highest value, but within milliseconds repassivation begins and the current density decreases. The next peak is due to film rupture and exposing new virgin material.

The current density can then be integrated into charge density $Q_{f}$ which is convenient to control the degradation of the CZM,

(3) $$Q_{f}=\int_{0}^{t_{f}}i_{0}{\left(\frac{t}{t_{0}}\right)}^{-m}dt$$

The slip-oxidation was introduced into the CZM using FE elements with the definition

(4) $$Q=\sum_{t=t_{0}}^{t}i_{t}\cdot \Delta t=\sum_{t=t_{0}}^{t}\left(i_{0}{\left(\frac{t}{t_{0}}\right)}^{-m(c)}\Delta t\right)\;\;t>t_{0}\;$$

where Δ*t* is the incremental time in the simulation. The charge density *Q* is only interesting after $t_{0}$ since the oxide starts to grow after $t_{0}$ and the model is only capturing the oxide growth. If the concentration is changed, Equation (4) needs to be used with an incremental time parameter Δ*t*, but this will force the time step to be low. If the concentration is constant over time, then *m(c)* = *m* and Equation (4) was changed from an piecewise linear summation to a single integration

(5) $$Q=-\frac{ti_{0}}{m-1}{\left(\frac{t_{0}}{t}\right)}^{m}+\frac{t_{0}i_{0}}{m-1}\;\;t>t_{0}$$

The parameter *m* was related to the passivity kinetic of the oxide film and dependent on the conductivity of the environment [4,31], which is deduced from the aggressive ions in the solutions diffused to the crack mouth.

**Figure 3:**
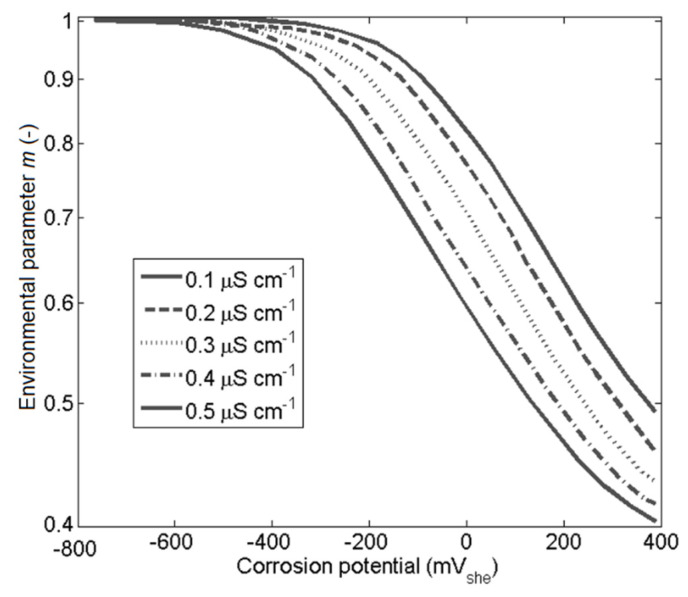
The different curves give the conductivity variations, Ford et al. [4].

The slip-oxidation model parameter *m* by Ford et al. [4,30] depends on the environmental properties, corrosion potential, and conductivity. The experimental data used was from Ford et al. [4], which defined the *m* parameter for the sensitized stainless steel 304 in corrosion potential from −600 to 400 mV_she_ and in conductivity ranging from 0.1 to 0.5 µs/cm, see Figure 3. The data were linearly interpolated or extrapolated for values not present. The relationship between concentration of ${\mathrm{Cl}}^{-}$ and conductivity in BWR was obtained from the BWR water chemistry guidelines [32].

The fracture time $t_{f}$ and oxide thickness at rupture was simulated by the model. The dislocation movements in the model were modelled as plastic strain $\varepsilon^{p}$ in the surround material.

**Figure 4:**
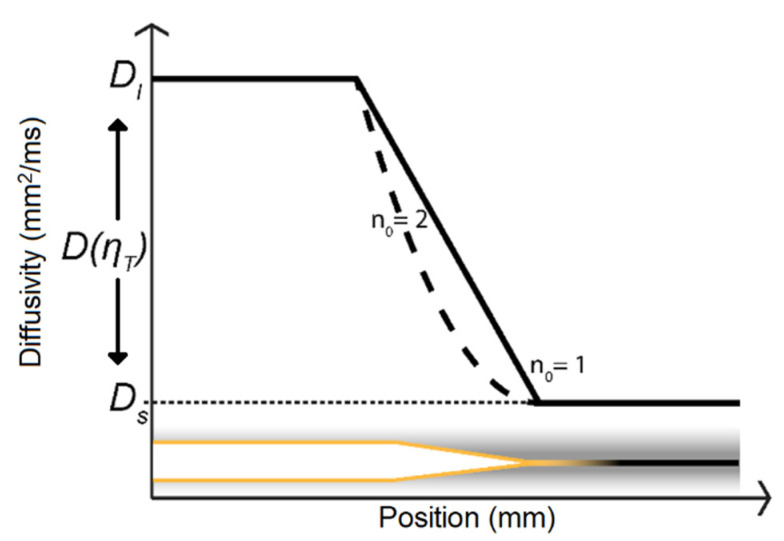
Diffusivity according to Equation (7) as a function of crack position. The solid line, $n_{0}=1$ gives a linear function while $n_{0}=2$, the dotted line, gives a quadratic shape.

The diffusivity was defined as

(6) $$\frac{\partial c}{\partial t}=D\left(\eta_{T}\right)\frac{\partial^{2}c}{\partial x^{2}}$$

where *c* is the concentration of aggressive ions that normally diffuse to the crack tip, ${\mathrm{Cl}}^{-}$ and ${\mathrm{SO}}_{4}^{2-}$ The crack was considered long. Therefore, the electromigration and convection were excluded. The time was defined as *t* and *x* was the spatial parameter along the crack path. The diffusivity parameter $D(\eta_{T})$ was defined as a function depending on the damage $\eta_{T}$ of the cohesive zone,

(7) $$D(\eta_{T})=(D_{l}-D_{s})\eta_{T}{}^{n_{0}}+D_{s}$$

where $D_{l}$ is the diffusivity in the damaged state of the cohesive zone, and $D_{s}$ is in the undamaged initial state. The *n*_0_ parameter is defined in the limit [1,∞], see Figure 4, the shape, $n_{0}=1$ is a linear function between $D_{l}$ and $D_{s}$, and was suitable for the investigated material 316L [10]. The diffusion was set up to create a moving boundary, by changing its diffusivity accordingly to the damaged state of the cohesive elements in Equation (7).

To implement the oxide film growth, an adaptive mesh was developed. The cohesive mesh was tied between bulk elements. The cohesive elements had the ability to adapt their length accordingly to Equation (4) and Faraday’s law

(8) $$t_{ox}=\frac{M}{z\rho F}Q$$

where $t_{ox}$ is the oxide thickness.

**Figure 5:**
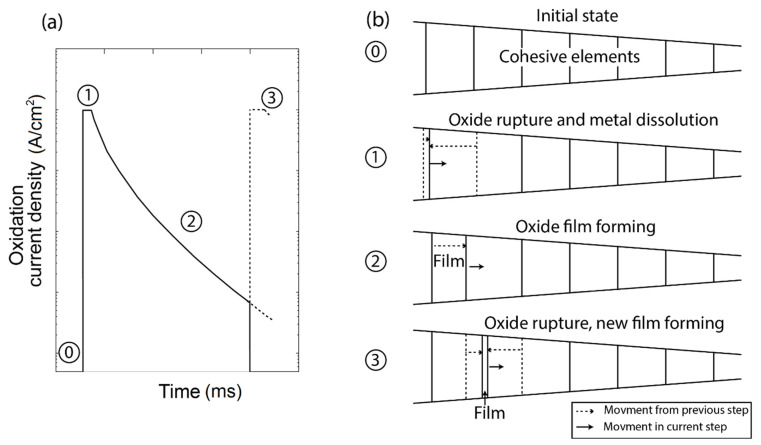
Node movement strategy for adaptive film growth: (**a**) schematic figure of oxide current density with respect to the time stamp (0–3) linked to (**b**) the displacements of the finite elements.

In Figure 5 the adaptive growth is shown in four steps. In the first initial step (0) in Figure 5a, no current density is present, and in Figure 5b the cohesive elements are in their initial arrangement. In step (1) the maximum current density is present, but the film has not started to grow. The movement is for the boundary cohesive elements, representing only dissolution. In step (2) the film starts to grow, therefore the boundary element nodes are locked and the elements start to grow. The thickness of the element was controlled by Equation (8), which gave the horizontal position of the moving nodes. In the next step (3), the cohesive element containing the oxide film became fully damaged due to the applied loads and its decreasing fracture energy which would correspond to a ruptured oxide film. At this instant, the ruptured oxide film element changed into liquid diffusivity element $D_{l}$, making the concentration move forward to interact with the next element. The process repeats itself but with the next element, creating crack advancement.

To satisfy a perpetual movement the length of the cohesive elements needed to be smaller than the thickness of the oxide. Eventually the movement created an oxide that interfered with the next cohesive element in front of it. The procedure was to force the element in front to follow the oxide and after oxide failure the cohesive element was disregarded. The routine was also allowed to jump over bulk element node connections by identifying them and leaping over them, see Appendix B for more details. The animations of the contour plots of the FE simulations are available in Sedlak [33], showing the adaptive node moving framework. For duplex models see Sedlak et al. [34].

## 3. Structural Model

**Figure 6:**
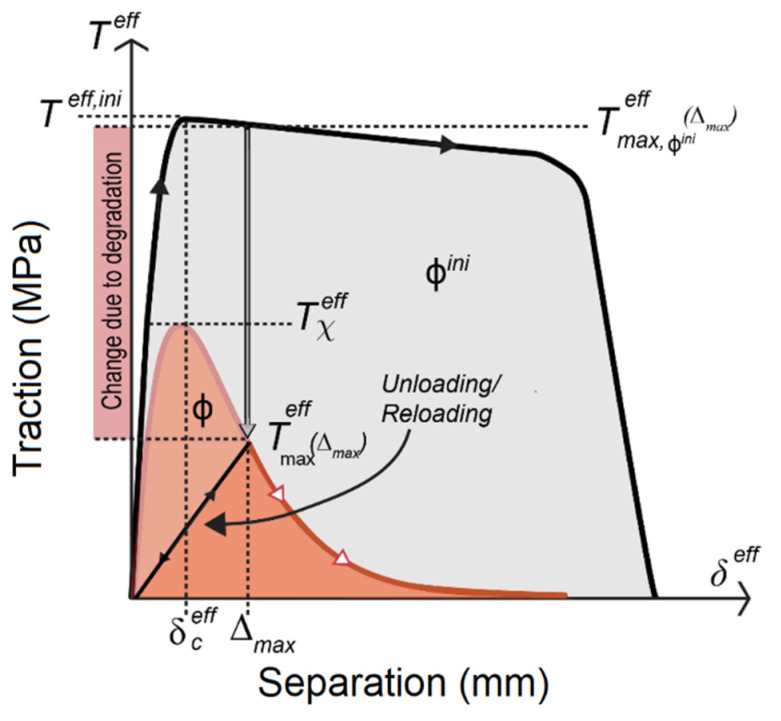
The effect of degradation on the TSL. The larger TSL represents the virgin material while the smaller TSL is the oxide.

The fracture mechanical model was set up in a cohesive element formulation. The cohesive element framework was based on the internal virtual work

(9) $$\delta \Pi_{\mathrm{int}}^{CZ}=\int_{\partial \Omega }\delta \Delta \cdot T_{CZ}dS$$

where $T_{CZ}$ is the cohesive traction and δΔ is the virtual separation. The model was included in the same user element subroutine, UEL in Abaqus (6.12, 2012, Dassault Systems, Johnston, RI, USA), as the electrochemical model, see Appendix C. The model was coupled through a segregated solution scheme. The cohesive element formulation utilized the potential based traction separation law (TSL) introduced by Park, Paulino, and Roesler (PPR) [21]. When the TSL was combined with the degradation model from Sedlak et al. [22], the TSL acquired the capability to change from a ductile to a brittle material, which here was from the austenitic stainless steel to its oxide products. The irreversibility was set up with the one parameter damage, introduced Ortiz and Pandolfi [35]. The state of the cohesive element was defined with the damage parameter $\eta_{T}$ with the limits [0,1]. $\eta_{T}=1$ stands for fully damage material,

(10) $$\eta_{T}=1-\frac{T_{max}^{eff}(\Delta_{\max})}{T_{\chi }^{eff}}$$

where $T_{max}^{eff}$ is the effective tractions at maximum separation and $T_{\chi }^{eff}$ is the current effective traction at the critical effective separation, see Figure 6. Due to the variation of softening shapes in the TSL, the damage parameter was defined in tractions instead of separations. The damage initiates when the separation has passed the effective initial separation $\delta_{c}^{eff}$ in Figure 6. The irreversibility introduced by Ortiz and Pandolfi [35] was implemented with an effective damage separation

(11) $$\Delta_{eff}=\sqrt{{\left(\Delta u_{1}\right)}^{2}+{\left(\Delta u_{n}\right)}^{2}}$$

where $\Delta u_{1}$ and $\Delta u_{n}$ are the current separations in the tangential and normal directions, respectively. During irreversibility Equation (11) submits to

(12) $$\Delta_{\max}\geq \Delta_{eff}$$

where $\Delta_{\max}$ is the maximum effective separation. During unloading and reloading a linear pattern was prescribed, see Figure 6.

## 4. Degradation

At IGSCC only intergranular fracture was considered, which means that the grain boundaries were dissolved by the electrochemical process and an oxide was formed. The fracture properties of the virgin material were changed to those of the oxide by the degradation process. The degradation process was introduced by changing the TSL shape of the cohesive elements, from the virgin grain boundary material to the fully oxidized material. The electrochemical part from Equation (8) describes the kinetics of the process and it was then coupled to the fracture energy Φ by,

(13) $$\begin{array}{l} \Phi_{n}=k_{}^{\Phi }Q^{-p}\\ \Phi_{1}=k_{}^{\Phi }Q^{-p} \end{array}$$

where $\Phi_{n}$ and $\Phi_{1}$ are the fracture energy in the normal and the tangential directions, respectively, $k^{\Phi }[>0,\infty )]$ and p[>0,∞)] are the proportion constants for the fracture energy Φ, and *Q* is the current density integrated over time. The relationship in Equation (13) follows a power law degradation process. The procedure to determine the parameters is described in Appendix A. The fracture energy has the limits $\Phi [\Phi^{full},\Phi^{ini}]$. The initial fracture $\Phi^{ini}$ was introduced for the virgin grain boundary material and the oxide fracture energy was denoted $\Phi^{full}$. Figure 6 illustrates the effect of degradation on the TSL. The black curve is for the initial grain boundary material and the red curve is for the oxide. The area enclosed by the TSL, after the initial separation $\delta_{c}^{eff}$, is the fracture energy. The change due to degradation is visualized by the *y*-axis, where an arrow shows the change in location between the two TSLs. Irreversibility is illustrated as the linear path between the origin and the maximum separation $\Delta_{\max}$ of the oxide TSL, defined in Equation (12). The loading/reloading path is followed during unloading and reloading. The change between different TSL shapes is controlled by the parameter *χ*, see Appendix D. The damage $\eta_{T}$ starts after the cohesive element reaches the separation $\delta_{c}^{eff}$ and any point of monotonic loading will always give the highest effective traction. However, if degradation is present prior to reaching $\delta_{c}^{eff}$ there is a higher effective traction $T_{}^{eff,ini}$ than the maximum effective traction and damage needs to be applied. This was overcome by storing the maximum traction $T_{max}^{eff}$ of the cohesive element and if the effective traction $T_{\chi }^{eff}$ of the degraded TSL becomes lower the damage is set to one,

(14) $$T_{max}^{eff}>T_{\chi }^{eff},\;\eta_{T}=1$$

and the maximum effective traction is defined as

(15) $$T_{max}^{eff}\geq T_{}^{eff}$$

## 5. Results

**Figure 7:**
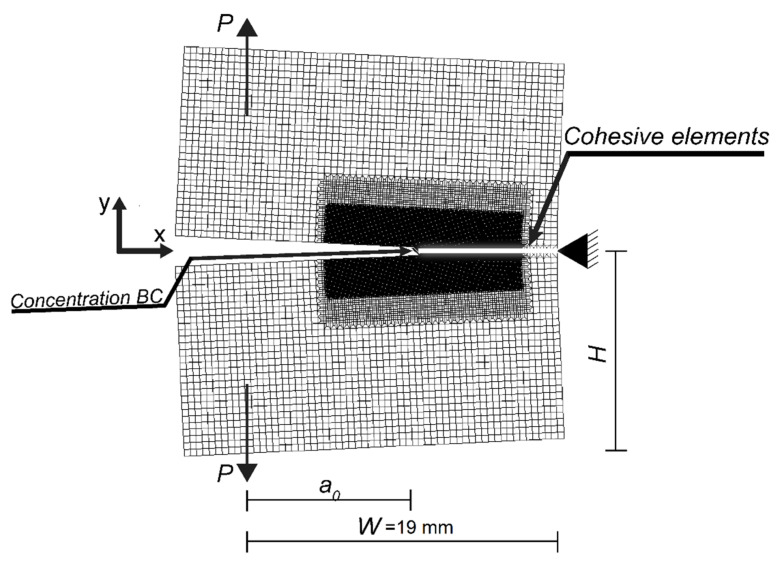
The compact tension specimen mesh and geometry. Where the concentration boundary condition (BC) is the initiation point of the SCC.

**Figure 8:**
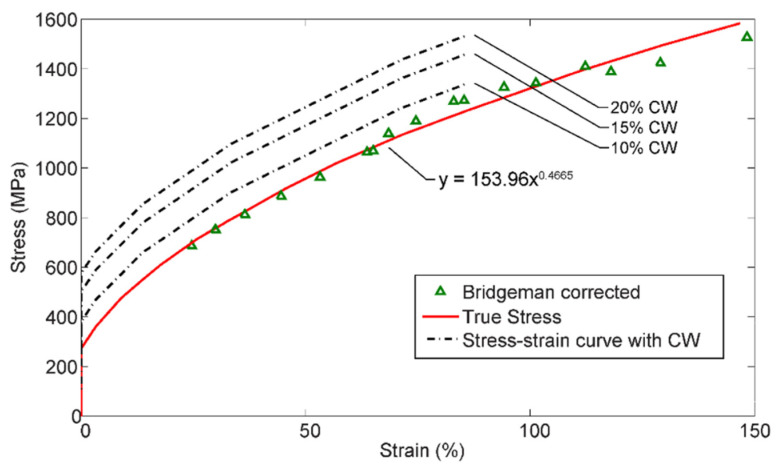
Stress-strain curve of the bulk material 316L from van Eeten and Nilsson [36]. Were the semi dotted lines are the effect of cold work (CW).

The model was set up using the subroutines in Abaqus and standalone Fortran 90 codes. All results were derived for the same material, austenitic stainless steel 316L, and compact tension (CT) geometry with the geometrical properties: initial crack length $a_{0}=25.3$ mm, thickness w=19 mm, half-height H=24.9 mm, and length W=40 mm, see Figure 7. The mesh was refined in three steps with the finest along the crack path. The model contained 36,176 plane strain elements for the bulk material. The side of smallest bulk elements, which was along the crack path, was 1 μm. Ninety-four cohesive elements were then placed at the interface between each bulk element along the crack path, see Figure 5. The cohesive element was then constrained with the Abaqus TIE subroutine to follow the bulk element along the crack path, but with the freedom to be manipulated by the adaptive oxide framework. The bulk material was formulated as elastic-plastic material with piecewise linear isotropic hardening, following the curve in Figure 8. The bulk material data was obtained from the experiments by van Eeten and Nilsson [36]. The figure includes the cold work (CW) at 10, 15, and 20% [37], which corresponds to the dotted lines in the stress-strain figure. The Young’s modulus was set to E=196 GPa, Poisson’s ratio was υ=0.27, and the undeformed yield stress was $\sigma_{Y0}=270$ MPa. The CT specimen was loaded to $K_{I}=15,\;27,\;\mathrm{or}\;37\;\mathrm{MPa}\sqrt{m}$, which corresponded to P=5, 9, or 12.4 kN. The concentration at the boundary was set to *c* = 10–90 ppb, which is typical for NWC [14].

The CZM parameters were divided in cohesive parameters, degradation parameter, diffusion parameters, and electrochemistry parameters. All the parameters were obtained at 288 °C, starting with the cohesive parameters for the virgin material. These were obtained from experimental results [38] and simulations [10]: $T_{n}^{ini}=T_{1}^{ini}=2500$ MPa, $\lambda_{n}^{ini}=\lambda_{1}^{ini}=0.1$, $\alpha^{ini}=\beta^{ini}=1.4$ and $\Phi_{n}^{ini}=\Phi_{1}^{ini}=400$ N/m. Iteration for the experimental SCC results by Ford et al. [4] gave the oxide parameters, $T_{n}^{full}=T_{1}^{full}=250$ MPa, $\lambda_{n}^{full}=\lambda_{1}^{full}=0.1$, $\alpha^{full}=\beta^{full}=10$, and $\Phi_{n}^{full}=\Phi_{1}^{full}=10$ N/mm. The degradation parameter *χ* was set to χ=3, which kept the ductile material behavior until 90% degradation, se Appendix D for TSL shapes with degradation parameter shapes χ=3.

There are two diffusion parameters, the diffusivity for the solid $D_{s}=1\times 10^{-18}$ mm^2^/ms, which was set to an arbitrary small value for zero diffusivity throughout the bulk material. The diffusivity in the liquid *D_l_* was obtained from Wilke-Change equation for diffusion coefficients in liquids [39]. It was set to the diffusivity of chlorides in water at 288 °C, giving $D_{l}=3.8\times 10^{-5}$ mm^2^/ms. The electrochemistry parameters were obtained from Shoji et al. [8]. The material parameters and constants in Faraday’s Law are presented in Appendix A. The last parameters $k_{}^{\Phi }=2.22$ N mm/(A ms) and p=0.749 defined the ratio between oxide degradation and charge density. They were determined by iteration on the SCC experiments by Ford et al. [4]. The parameters are listed in Appendix A.

The model was verified against experiments in two mechanical aspects. The yield stress was changed and the stress intensity factor was changed. It was also studied during changes of conductivity due to chloride changes and corrosion potential variations.

### 5.1. Yield Strength

**Figure 9:**
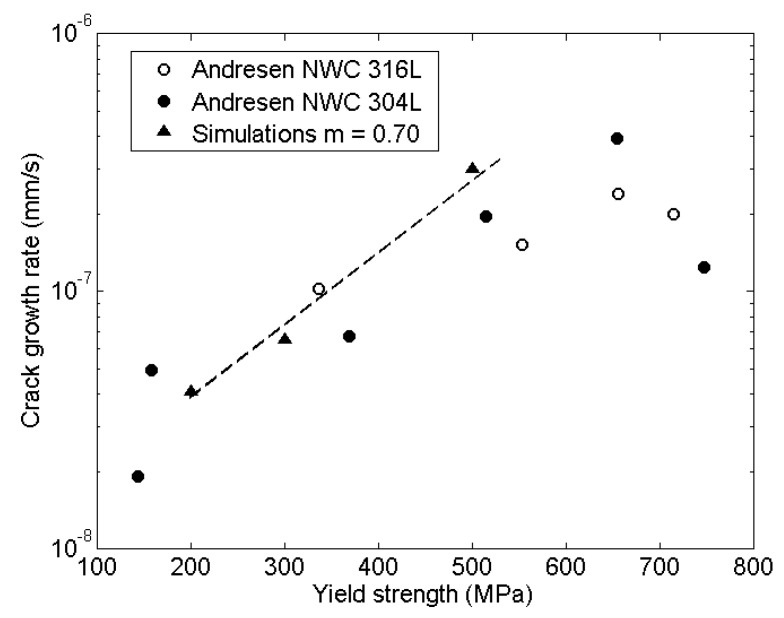
Effect of *σ*_Y_ on crack growth rate. The experiments by Andresen [40] were conducted in normal water chemistry (NWC).

Cold work was simulated by changing the yield stress of the bulk material according to $\sigma_{Y}$ = 200, 300, 500 MPa in normal water chemistry (NWC). The environmental parameter was set to fit the $\sigma_{Y}$ = 300 MPa result in the experiments by Andresen [40] and $K_{I}$ = 27 MPam^1/2^. The results for varied $\sigma_{Y}$ are compared to the experiments in Figure 9, showing good agreement. The triangles present the simulation results, the circles are the experimental results and the dotted line is a computational fit. The agreement between experimental results for 316L and 304L confirms the validity to use the same mechanical properties for both materials.

**Figure 10:**
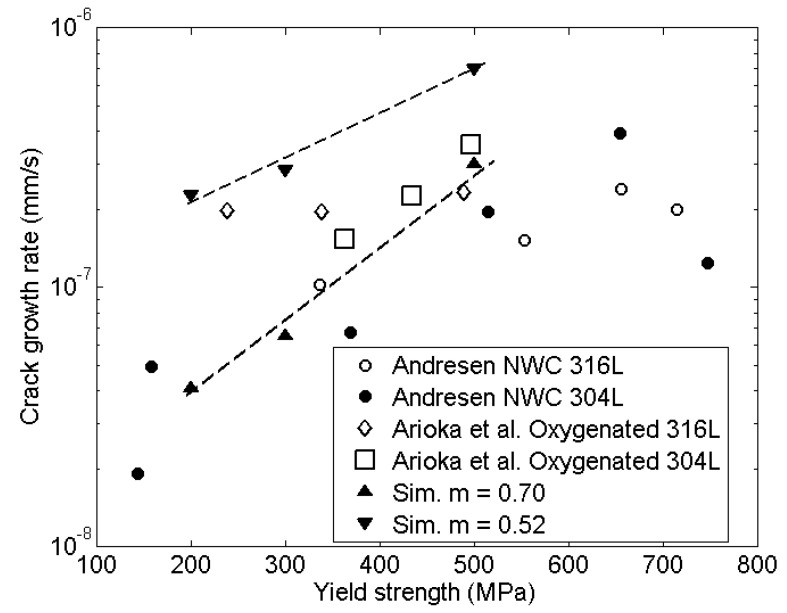
Effect of yield strength and environment on crack growth rate.

Figure 10 shows the influence of environment by changing the environmental parameter between *m =* 0.52 and 0.70. This corresponds to 0.3 µs/cm, the corrosion potential +150 mV_she_ and 0.3 µs/cm and the corrosion potential 0 mV_she_, respectively. The results where compared to data generated by Arioka et al. [41] in oxidizing environment. The squares are the experimental results from Arioka et al. for oxidizing environment for the material 304L and the diamonds for 316L. The circles are results from Andresen [40], where the full circles are 304L and the rest are for 316L. The triangles represent the simulated results. The results for *m* = 0.70, triangles pointing up, fitted well as shown in Figure 10, but the result for *m* = 0.52 with higher corrosion potential showed a positive trend but overestimated the experimental results from Arioka et al. [41].

### 5.2. Stress Intensity Factor

**Figure 11:**
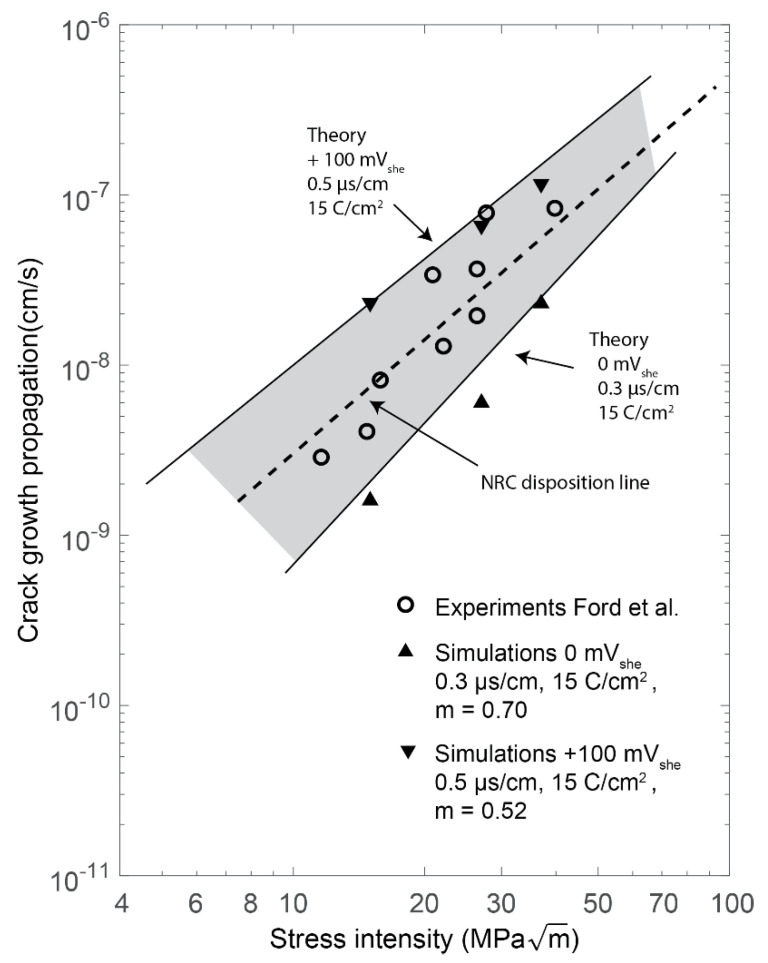
Effect of stress intensity on crack propagation in two environments. The NRC line is the regulatory relationship used for structural integrity evaluations [43].

The crack growth rate due to some stress intensity factor values was studied in different environments. The crack tip was exposed to $K_{I}$ = 15, 27, or $37\;\mathrm{MPa}\sqrt{m}$. The stress intensity factor *K_I_* was calculated at the initial crack length $a_{0}$, giving the force *P* acting on the force BC. The force and the environmental parameter *m* were kept constant throughout each simulation. The results were compared to the results by Ford et al. [4]. The conditions in the experiment were sensitized 304 stainless steel in LWR environment. For the simulations, the mechanical material data from 316L was used since the mechanical data for 304 and 316L are similar, and the IGSCC results by Andresen [40] shows equal behavior with respect to $\sigma_{Y}$. Two environments were studied. In the first environment, the conductivity was 0.5 µs/cm and the corrosion potential was +100 mV_she_ and for the second environment, the conductivity was 0.3 µs/cm and the corrosion potential was 0 mV_she_, which gave the environmental parameter *m =* 0.52 and 0.70 respectively from experiments by Ford et al. [4], se Appendix A. The results from the simulation are shown as triangles in Figure 11. The upward pointing triangles shows the results for the crack growth rate in the environment corresponding to *m =* 0.52 and the downward facing triangles in the less corrosive environment with *m =* 0.70. The experimental data, shown as circles, are from Ford et al. [4], the dotted line and the semi-dotted lines are two theoretical curves are also by Ford et al. [4] from the slip-oxidation model [4,42]. The two theoretical curves are computed for the conductivity 0.5 and 0.3 µs/cm and the corrosion potential +100 and 0 mV_she_, which are the same environments that were simulated. The full line is the NRC regulatory relationship used for structural integrity evaluations [43]. The simulation with *m* = 0.70 which corresponded to the lower bound theory fitted well and the simulation with *m* = 0.52 which corresponded with the upper bound also fitted well.

**Figure 12:**
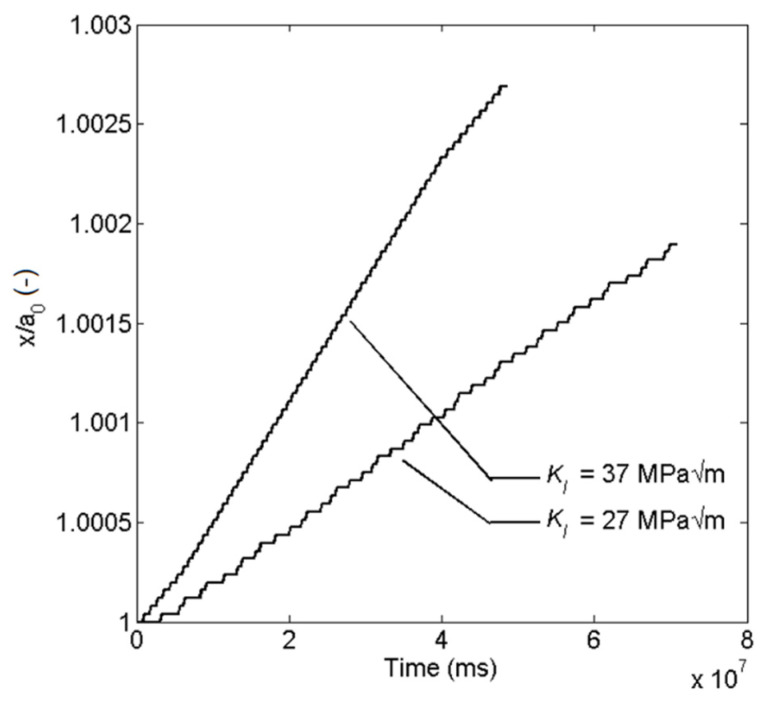
Crack tip position with respect to time.

The crack growth in Figure 12 is for $K_{I}$ = 27 and $37\;\mathrm{MPa}\sqrt{m}$ with the environmental parameter *m =* 0.52. The step like curves are the result from the cyclic film rupture process.

**Figure 13:**
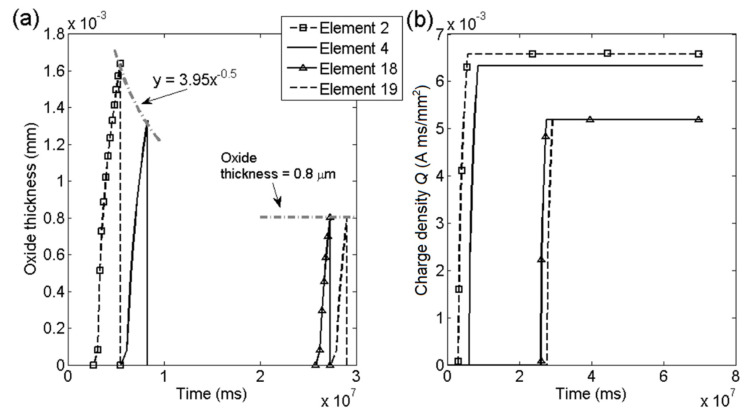
Effect of $K_{I}$ = 27 $\mathrm{MPa}\sqrt{m}$: (**a**) Oxide thickness and (**b**) charge density *Q* in elements.

**Figure 14:**
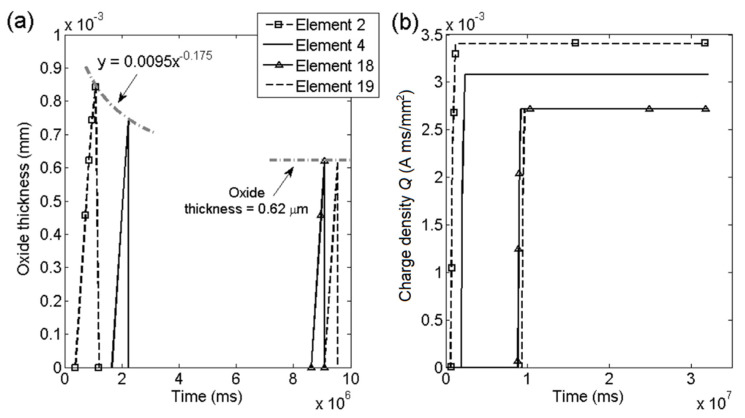
Effect of $K_{I}$ = $37\;\mathrm{MPa}\sqrt{m}$: (**a**) Oxide thickness and (**b**) charge density *Q* in elements.

Figure 13 shows the oxide thickness and oxide density for four cohesive elements at $K_{I}$ = 27 $\mathrm{MPa}\sqrt{m}$ and Figure 14 shows the same response for the same elements at $K_{I}$ = 37 $\mathrm{MPa}\sqrt{m}$. The change in stress intensity factor was obtained by changing the force boundary condition. The first two element chosen were affected by the initial plastic zone and therefore shows a higher result, the two subsequent elements 18,19 showed stable result for both applied boundary conditions. At elements 18,19 for both stress intensities the oxide thickness converged into similar results over time. The lower stress intensity factor $K_{I}$ = 27 $\mathrm{MPa}\sqrt{m}$ exhibited thicker oxide than for the higher stress intensity factor. The results show that higher crack growth rates are obtained by thinner oxides and more frequent oxide ruptures, which was expected based on Equation (5). In Figure 13b and Figure 14b the charge density was shown for the same elements, showing fast growth during oxide growth and no increase after oxide rupture. The charge density shows similar value for elements 18, 19 for both stress intensity cases. The animations of the contour plots of the FE simulations are available in Sedlak [33]. The animations show the two simulations illustrated in Figure 11 and Figure 12 with $K_{I}$ = 27 and 37 $\mathrm{MPa}\sqrt{m}$. The contour plots were evaluated in, concentration, stresses in *y*-direction and displacement.

## 6. Conclusions

An IGSCC model was developed. It incorporated the slip-oxidation model [4] with adaptive oxide thickness and the cohesive zone fracture model. The model utilized the enhanced cohesive degradation formulation by Sedlak et al. [22], the degradation was traction based [10], and an adaptive method to move and tie the nodes of the cohesive elements to the bulk material was developed and implemented to create a continuously growing oxide film.

- The cyclic physics of the slip oxidation model was replicated. In the model, the thickness of the oxide was taken into consideration as the physical length of the cohesive element. The cyclic process was modelled with oxide film growth, oxide rupture, and re-passivation.
- The model shows good agreements with experiments in the literature for changes in stress intensity factor, yield stress representing cold work, and environmental factors such as conductivity and corrosion potential.
- When comparing the stress intensity factor simulations to the theoretical upper limit, the simulations gave an underestimation by a mean factor of 1.15. The lower limit was underestimated by a factor of 2.61. The mean deviation during all stress intensity simulations was calculated to $8.9\times 10^{-9}\;\mathrm{cm}/s$.
- Cold works simulations gave good agreement also with experiments in oxidizing environment. In the experimental data the non-oxidized to oxidized environment increased the crack growth rate by a factor of 4.3 at yield strength 370 MPa and 1.8 at 490 MPa. The simulations gave an increase of 4 at yield strength 370 MPa and 2.3 at 490 MPa.
- The model was computational and cost effective for predicting IGSCC, which is useful for optimization situations.

## Figures and Tables

**Figure A1:**
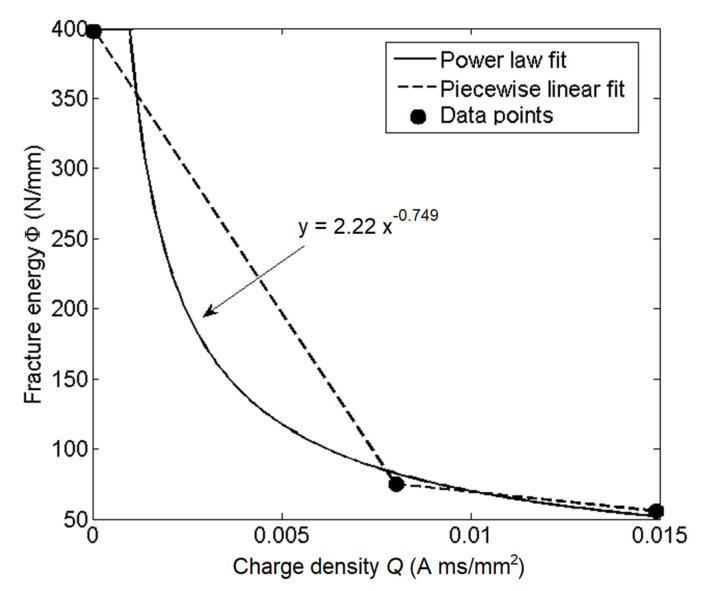
The degradation power law parameters are extracted from the data points from the simulations at different stress intensity factors.

**Figure A2:**
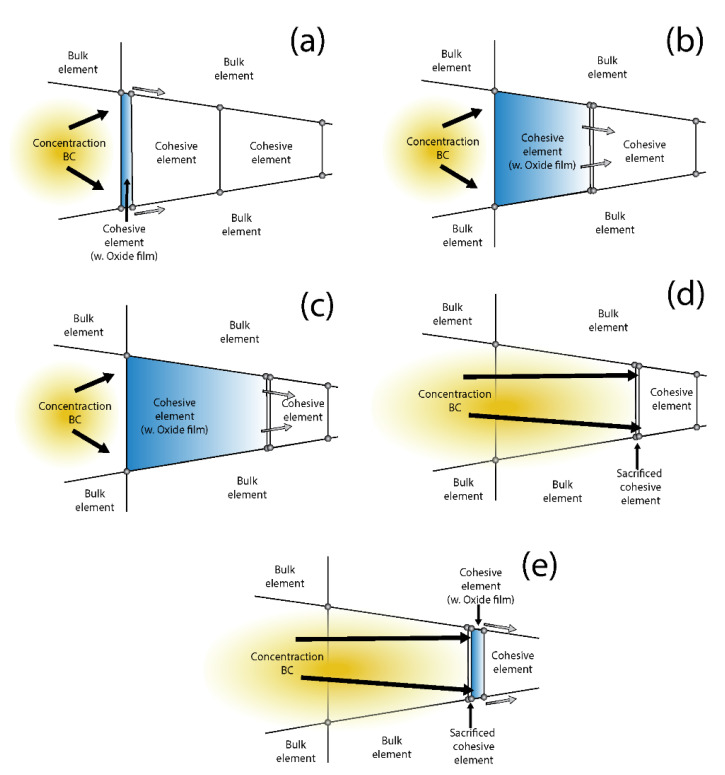
The adaptive mesh, oxide contact movement: **(a)** start of oxide growth, **(b)** continuous oxide growth, **(c)** oxide growth past the adjacent cohesive element. **(d)** Rupture oxide **(e)** new oxide forming one cohesive element scarified.

**Figure A3:**
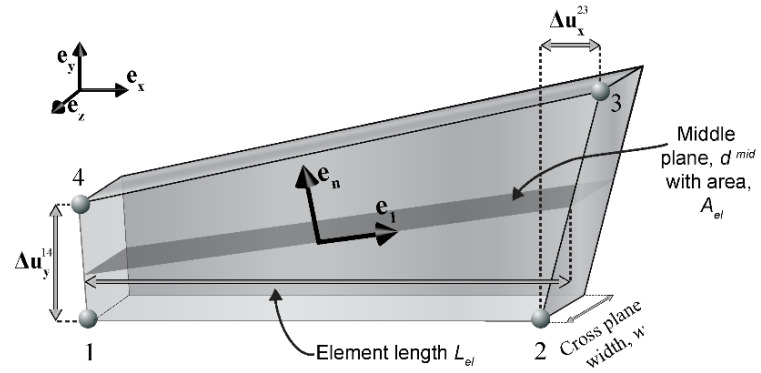
A cohesive element.

**Figure A4:**
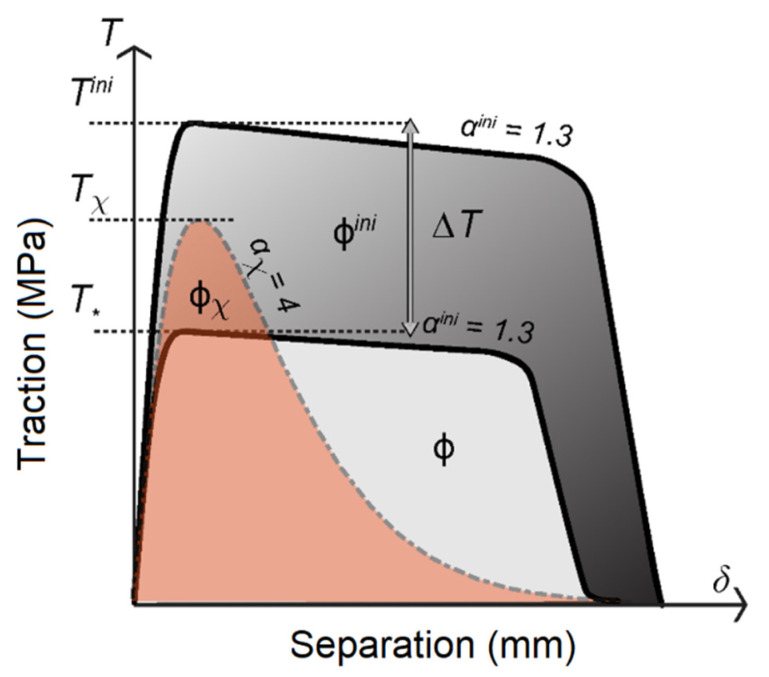
TSL during a degradation step. The upper convex TSL is the initial TSL, the lower convex TSL is intermediate after degradation but before shape change, and the concave TSL is after implementing the shape changes. Note that Φχ = Φ.

**Figure A5:**
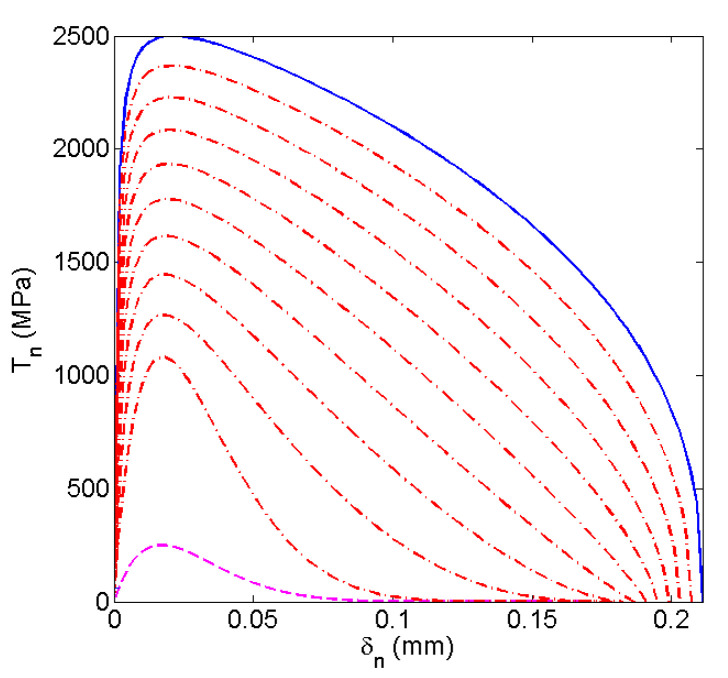
The degradation effect on the TSL with shape changing factor χ=3. The dashed curves are the degraded intermediate curves except for the last one which is the fully degraded TSL, the solid curve is the virgin bulk material TSL.

## Data Availability

The raw/processed data required to reproduce these findings cannot be shared at this time as the data also forms part of an ongoing study. A part of the processed data is available to download from Mendeley Data [33].

## Nomenclature

A	model parameter (C mm^3^)
$a,a_{0}$	crack length, initial crack length (mm)
c	concentration (ppb)
$D_{},D_{l},D_{s}$	diffusivity, in liquid and in solid, respectively (mm^2^/ms)
E	Young’s modulus, (GPa)
F	Faraday’s constant (C/mol)
H	half height of specimen (mm)
$i_{0},i_{t},i$	initial-, slope current density, current density (A/mm^2^)
*K_I_*	stress intensity factor (MPa$\sqrt{m}$)
$k^{\Phi },k_{{}^{1}}^{\Phi },k_{{}^{2}}^{\Phi }$	energy proportion constants (N mm/(A ms))
M	Atomic weight (g/Mol)
m	environmental parameter
$n_{0}$	process parameter, (MPa mm^2^/g)
P	force (kN)
p	energy proportion constant
$Q,Q_{f}$	charge density, charge density for one cycle (A ms/mm^2^)
$T_{}^{eff},T_{\max}^{eff},T_{}^{eff,ini}$	effective traction, current maximum traction and initial traction, respectively (MPa)
$T_{n}^{ini},T_{1}^{ini}$	normal and tangential initial tractions (MPa)
$T_{n}^{full},T_{1}^{full}$	normal and tangential fully degraded TSL tractions (MPa)
$T_{\chi }^{eff},T_{\chi ,n}^{},T_{\chi ,1}^{}$	current tractions: effective, normal and tangential, respectively (MPa)
$T_{CZ}$	cohesive traction (MPa)
t	time (ms)
$t_{0},t_{f}$	time for repassivation, time between ruptures (ms)
$t_{ox}$	oxide thickness (mm)
W	length of CT-specimen (mm)
w	thickness (mm)
x	spatial coordinate (mm)
y	fitted equation for oxide thickness (mm)
$z_{}$	electron exchanged
$\alpha^{ini},\alpha^{full}$	initial and fully degraded normal softening parameter
$\beta^{ini},\beta^{full}$	initial and fully degraded tangential softening parameter
γ	constant (1/s)
$\Delta_{eff}$	cohesive element separation (mm)
$\Delta_{\max}$	maximum effective separation (mm)
Δt	incremental time step (ms)
$\Delta u_{n},\Delta u_{1}$	normal and tangential current separation (mm)
$\delta_{c}^{eff}$	critical effective separation (mm)
$\delta^{eff}$	effective separation (mm)
δΔ	virtual separation (mm)
$\delta \Pi_{\mathrm{int}}^{CZ}$	internal virtual work (Nmm)
$\varepsilon_{f}$	surface film rupture strain
${\dot{\varepsilon }}_{ct}$	crack tip strain rate (1/s)
$\eta_{T}$	damage parameter
$\lambda_{n}^{ini},\lambda_{1}^{ini}$	normal and tangential initial slope indicators
$\lambda_{n}^{full},\lambda_{1}^{full}$	normal and tangential fully degraded slope indicators
ρ	mass density (g/mm^3^)
$\sigma_{Y},\sigma_{Y0}$	yield stress (MPa)
υ	Poisson’s ratio
χ	degradation parameter
$\Phi^{k}$	fracture energy at the crossing point (N/mm)
$\Phi_{n}^{},\Phi_{1}^{}$	normal and tangential fracture energies (N/mm)
$\Phi_{n}^{ini},\Phi_{1}^{ini}$	normal and tangential initial fracture energies (N/mm)
$\Phi_{n}^{full},\Phi_{1}^{full}$	normal and tangential fully degraded fracture energies (N/mm)

## Appendix A

### Appenidx A.1. Environment Parameters

Table A1 presents the parameters used in Faraday’s Equation (1).

**Table A1:** Parameters used in the Faraday Equation (1) from Shoji et al. [8].

Parameters	Standard Value
Atomic weight, *M* (g/Mol)	55.382
Oxidation current density, *i*_0_ (A/cm^2^)	0.12
Number of electrons exchanged, *z*	2.67
Faraday’s constant, F (C/mol)	96,500
Mass density, *ρ* (g/mm^3^)	0.00786

### Appenidx A.2. Degradation Parameters

The degradation parameters $k_{}^{\Phi }$ and p can be found by running the simulation at two different stress intensity factors and then fitting the power law. The power law can also be changed to a piecewise linear function. A piecewise linear function defined in three points is a bilinear function,

(A1) $$\begin{array}{ll} \Phi_{}=\Phi_{}^{ini}-k_{1}^{\Phi }Q^{} & \Phi <\Phi^{k}\\ \Phi_{}=\left(\Phi_{}^{k}+k_{1}^{\Phi }k_{2}^{\Phi }\left(\frac{\Phi_{}^{ini}-\Phi^{k}}{k_{1}^{\Phi^{}}}\right)\right)-(k_{1}^{\Phi }k_{2}^{\Phi }Q^{})\; & \Phi \geq \Phi^{k} \end{array}$$

This function will be needed parameters $k_{1}^{\Phi }$, $k_{2}^{\Phi }$, and $\Phi^{k}$. The parameters were obtained by running simulations at two different stages and knowing the initial stage giving, $k_{1}^{\Phi }=1.9\times 10^{5}$ N mm/(A ms), which gave the initial slope in the bilinear function and $k_{2}^{\Phi }=0.008$ which is a multiplier for the slope of the second line, giving the slope as $k_{1}^{\Phi }k_{2}^{\Phi }$. The last parameter sets the fracture energy at the crossing point $\Phi^{k}=75$ N/m. If the three point were fitted for a power law, then $k_{}^{\Phi }=2.22$ N mm/(A ms) and p=0.749 were found. Both the bi-linear and power law function can be used in the model, depending on the best fit for the real-life application.

## Appendix B

The model was set up in several Abaqus subroutines, user element UEL, multi-point constraints MPC, distributed load DLOAD, prescribed displacement DISP and some global subroutines not defined by Abaqus with global parameters/variables. The main part was introduced in the user element UEL as a cohesive element containing the PPR model, the degradation patch, the diffusion and the adaptive move. The multi-point connectivity subroutine MPC contained the tie contact of the cohesive elements and executed the movement of the nodes for the adaptive framework. The distributed load subroutine DLOAD applied the smooth initial line load on the boundary condition (BC) elements to create the correct load. The user displacement subroutine DISP was used to set the initial conditions for the concentration BC. The other subroutines not issued by Abaqus were used to read input and create the FE mesh.

The adaptation was set to run at every iteration. This forced the oxide to grow at every step, showing the actual oxide size by the relative node displacement. Initially the mesh of the cohesive elements was distributed uniformly along the expected crack path. As soon as the routine detected an oxide growth over a certain length (0.01 µm) the upper and lower nodes of the cohesive element were translated to the location of the tip of the oxide film. The result of the initial movement created the oxide film. This movement was repeated after every film brakeage. Both Figure 5 and Figure A2 show the results of the movement. However, sometimes the film would not break before running into the neighboring cohesive element. If this happened the second element was pushed forward Figure A2b,c until the cohesive element with the oxide film breaks. The moved element was sacrificed, Figure A2d, and the neighboring cohesive element took over the oxide film forming process in Figure A2e.

## Appendix C

### Appenidx C.1. Diffusion Element Formulation

Equation (6) was reworked into the weak form. The weighted integral and divergence theorem was applied and the shape functions $N_{i}$ were used. The diffusion Equation (6) was discretize giving the semi-discrete matrix system

(A2) $$M\frac{\partial c_{e}}{\partial t}+Kc_{e}=0$$

where **M** is the element mass concentration matrix and **K** is the element diffusivity matrix, defined as

(A3) $$M=A\int_{x}\rho N^{T}Ndx$$

(A4) $$K=A\int_{x}D(\Delta_{eff})B^{T}Bdx$$

where **B** are the derivatives of the shape functions and $A=w\Delta u^{mid}(\xi =1)$ is the surface area. The opening of the cohesive element is $\Delta u^{mid}$ and w is the cross-plane width, see Figure A3. To discretize Equation (A2) in time, backward Euler iteration scheme was used

(A5) $$\frac{\partial c_{e}}{\partial t}=\frac{c_{e}^{\Delta t+t}-c_{e}^{t}}{\Delta t}$$

Abaqus needs the Jacaobian (stiffness) and the internal force vector defined as

(A6) $$f_{diff}^{el}=[A\int_{x}\rho N^{T}Ndx+\Delta tA\int_{x}D(\Delta_{eff})B^{T}Bdx]c_{e}^{\Delta t+t}-A\int_{x}\rho N^{T}Ndxc_{e}^{t}$$

(A7) $$\begin{array}{l} K_{diff}^{el}=A\int_{x}\rho N^{T}Ndx+\Delta tA\int_{x}D(\Delta_{eff})B^{T}Bdx \end{array}$$

### Appenidx C.2. Structural Element Formulation

The internal force matrix for the structural element

(A8) $$f_{N}^{e}=w\int_{L_{el}}{\hat{N}}^{T}[{\hat{e}}_{1},{\hat{e}}_{n}]t_{}^{mid}dl$$

where $\hat{N}$ are the separation shape functions defined as $\hat{N}=Ν\Phi$,

(A9) $$\Phi =\left[I_{4\times 4}|\begin{array}{cc} 0_{2\times 2} & \times_{2\times 2}\\ I_{2\times 2} & 0_{2\times 2} \end{array}\right]$$

In Equation (A8) *w* is the cross-plane width, $L_{el}$ is the length of the middle plane in the element, $t_{}^{mid}$ is the local traction vector obtained from the TSL, ${\hat{e}}_{1}$ is the mid-plane normalized direction vector tangential to the mid-plane, and ${\hat{e}}_{n}$ is in the normal direction to the mid-plane, see Figure A3. In Equation (A9) $I_{4\times 4}$ and $I_{2\times 2}$ are the identity matrices. The algorithmic element stiffens matrix was defined as

(A10) $$K^{e}=\int_{\partial \Omega_{mid}^{CZ,e}}{\left(N\Phi \right)}^{T}\Theta (n)\frac{\partial t^{mid}}{\partial d_{e}}ds+\int_{\partial \Omega_{mid}^{CZ,e}}{\left(N\Phi \right)}^{T}\frac{\partial \Theta (n)}{\partial d_{e}}t^{mid}ds$$

where the first integral is the material stiffness and the second integral is the geometrical stiffness. The derivation of Equation (A10) is detailed in Sedlak et al. [22]. $d_{e}$ are the displacements.

## Appendix D

The degradation was controlled by reducing the fracture energy, which could be done in two ways, by changing the maximum cohesive strength *T*^eff^ or by changing the softening shape through the parameter *α* in the TSL [14]. For a given energy reduction, degradation could favor a fast change to a brittle material by keeping a high maximum cohesive strength, see the dashed concave TSL line in Figure A4, or remaining ductile, see the lower convex solid TSL in Figure A4. The degradation process was introduced in two numerical steps. The fracture energy Φ^ini^ was first reduced from the initial TSL, the top solid curve in Figure A4 to the lower solid curve with fracture energy Φ and strength $T_{*}$ in the figure. Then the TSL softening parameter *α* was adjusted for the degraded maximum cohesive strength *T*_χ_. The control parameter χ was defined as

(A11) $$\chi =\frac{T^{ini}-T_{\chi }}{T^{ini}-T_{*}}$$

The TSL with shape changing factor was obtained by fitting to experimental results [4] and following the procedure outlined by Sedlak et al. [22]. The degradation shapes are shown in Figure 7.
